# *Drosophila* as a Model System to Investigate the Effects of Mitochondrial Variation on Innate Immunity

**DOI:** 10.3389/fimmu.2020.00521

**Published:** 2020-03-25

**Authors:** Tiina S. Salminen, Pedro F. Vale

**Affiliations:** ^1^School of Biological Sciences, Institute of Evolutionary Biology, The University of Edinburgh, Edinburgh, United Kingdom; ^2^Faculty of Medicine and Health Technology, Tampere University, Tampere, Finland

**Keywords:** *Drosophila melanogaster*, cybrid, infection, innate immunity, mitochondria, mtDNA, oxidative phosphorylation, reactive oxygen species

## Abstract

Understanding why the response to infection varies between individuals remains one of the major challenges in immunology and infection biology. A substantial proportion of this heterogeneity can be explained by individual genetic differences which result in variable immune responses, and there are many examples of polymorphisms in nuclear-encoded genes that alter immunocompetence. However, how immunity is affected by genetic polymorphism in an additional genome, inherited maternally inside mitochondria (mtDNA), has been relatively understudied. Mitochondria are increasingly recognized as important mediators of innate immune responses, not only because they are the main source of energy required for costly immune responses, but also because by-products of mitochondrial metabolism, such as reactive oxygen species (ROS), may have direct microbicidal action. Yet, it is currently unclear how naturally occurring variation in mtDNA contributes to heterogeneity in infection outcomes. In this review article, we describe potential sources of variation in mitochondrial function that may arise due to mutations in vital nuclear and mitochondrial components of energy production or due to a disruption in mito-nuclear crosstalk. We then highlight how these changes in mitochondrial function can impact immune responses, focusing on their effects on ATP- and ROS-generating pathways, as well as immune signaling. Finally, we outline how being a powerful and genetically tractable model of infection, immunity and mitochondrial genetics makes the fruit fly *Drosophila melanogaster* ideally suited to dissect mitochondrial effects on innate immune responses to infection.

## Introduction

Understanding why individuals vary in their response to infection is one of the major challenges in immunology (1, 2). This variation may arise from differences in host age (3), sex (4), nutrition or environmental stressors (5), and genetic variation present in immune related genes (2). Experimental immunology – mainly in model systems such as mice, zebra fish and fruit flies – has been successful in identifying the major immune pathways (*Drosophila* innate immunity summarized in Box 1) (6–8). Quantitative genetic and genomic approaches have identified polymorphisms in genes underlying these mechanisms, and these explain some of the variation in infection outcomes (9–12). While most of this work has focused on genetic variation in the nuclear genome, metazoan organisms have an additional genome, inherited maternally inside mitochondria (mtDNA). More than functioning as the powerhouses of the cell, a growing body of work in the last decade has shown that mitochondria play an important role in inflammation and immunity and contribute to the host response to infection (13–18).

**BOX 1.**

*Drosophila* innate immunity in a nutshell. *Drosophila* has been extensively utilized as a model system for innate immunity and it has led to many breakthrough in immunity field (6, 114, 115). *Drosophila* does not possess acquired/adaptive immunity and it relies on humoral and cell-mediated innate immunity for its defense against pathogens, such as bacteria, viruses, fungi, and parasites. Immune mechanisms against these invaders include activation of appropriate signal transduction pathways depending on the invading microbe, involving production of antimicrobial peptides (AMPs), phagocytosis of microbes, wound closure, and a melanization cascade involved in the encapsulation of foreign elements. Similar first-line innate immune defense mechanisms can be found from plants to humans.

**Humoral Innate Immunity:** In *Drosophila*, the humoral innate immune response to bacterial pathogens is characterized by the production and release of a cocktail of AMPs into the hemolymph. This response is driven by two evolutionarily conserved and largely independent pathways, Immune deficiency (IMD) and Toll pathways (116). The Toll pathway is induced by bacteria containing LYS-type peptidoglycan in their cell walls (mainly Gram-positive bacteria), while the IMD pathway is induced by DAP-type peptidoglycan (mainly Gram-negative) bacteria. These pathways culminate in the translocation of NF-κB dimers to the nucleus leading to infection-specific upregulation of AMPs targeted to clear the infection (117–119). The response to viral pathogens replicating within the host cells involves both the Janus kinase/signal transducers and activators of transcription (JAK-STAT) pathway (118, 120), RNA interference (RNAi) and antiviral effector molecules (121, 122). Viral infections involve cell-mediated responses like apoptosis and autophagy and humoral responses such as the expression of anti-viral genes, some of which overlap with genes induced upon bacterial and fungal infections, indicating the involvement of the NF-κB signaling upon viral infections. The response to fungal invaders includes both humoral and cellular arms of immunity and involves the expression of AMPs mainly via the Toll pathway.

**Cell-Mediated Innate Immunity:** In *Drosophila*, the cell-mediated innate immune system consists of hemocytes (blood cells) and is induced by epithelial damage and detection of foreign particles in the hemocoel. Hemocytes function in sealing of epithelial wounds, encapsulating and terminating parasites and engulfing apoptotic corpses [reviewed in (123)]. In *Drosophila* there are three major lineages of hemocytes: plasmatocytes (phagocytic), crystal cells (melanization) and lamellocytes (encapsulation). Plasmatocytes comprise the majority of the circulating hemocyte population and are responsible for the engulfment of small particles, participate in the encapsulation of foreign material and are able to trigger the systemic humoral immune response to secrete AMPs. Crystal cells usually make up less than 5% of the larval circulating hemocytes. Crystal cells contain prophenol oxidase which active form phenol oxidase is involved in the melanization cascade when the crystal cells rupture in response to immune activation (123). In uninfected larvae, the lamellocytes can be present in small numbers in the late third instar stage, otherwise healthy larvae do not contain them. Lamellocytes are produced upon invasion of parasitoid wasps and they form a multilayer capsule around the invading parasitic egg, with the help of plasmatocytes and crystal cells (124). Eventually the capsule is melanized and elevated levels of ROS terminate the intruder (125).

Here, we propose that the fruit fly (*Drosophila melanogaster)* offers an ideal model system to investigate the role of mitochondrial variation and mito-nuclear crosstalk in innate immunity. We start by discussing the sources of variation in mitochondrial function, using examples of mutations of nDNA and mtDNA encoded genes that have been shown to affect organismal phenotypes through changes in mitochondrial metabolism and signaling. This is followed by emphasizing the emerging role of mitochondria in immune responses through mitochondrial metabolites and by-products of mitochondrial metabolism, such as ROS. Finally, we describe methodology to investigate the role of mito-nuclear crosstalk and mtDNA variation in immunity in *Drosophila*. We emphasize how the use of cytoplasmic hybrid (cybrid) models allows to distinguish the effect of mtDNA variation from that arising from the nuclear genome. We conclude by highlighting the benefits of the cybrid model to further our understanding of mito-nuclear effects on heterogenous immune responses.

## Sources of Mitochondrial Variation

Mitochondrial function depends on ∼1200 – 1500 proteins, the majority of which are encoded by the nuclear genome and transported to the mitochondria (19). Cellular energy production relies on mitochondria to produce ATP via oxidative phosphorylation (OXPHOS). OXPHOS requires the coordinated function of multiple protein subunits encoded by both the nuclear and mitochondrial genomes (nDNA and mtDNA, respectively – Box 2), and therefore both anterograde (from nucleus to mitochondria) and retrograde (from mitochondria to nucleus) signaling is required for optimal mitochondrial function. Mitochondrial variation arising from either nDNA or mtDNA can affect the transcription and translation of the mitochondrial proteins, signaling between the two genomes and through changes in the direct physical interactions among the OXPHOS components originating from the two genomes, ultimately affecting the function of mitochondria. Mitochondrial variation shows multiple mode of inheritance. When this variation originates from mtDNA it is maternally inherited and has a potential to become heteroplasmic even within mitochondria, and when originating from nuclear genome it can be X-linked, autosomal dominant, autosomal recessive or *de novo*. Here, we discuss potential sources of mitochondrial variation with examples of known nuclear and mitochondrial mutations that could also lead to variation in immune responses.

**BOX 2.**

**FIGURE 1:**
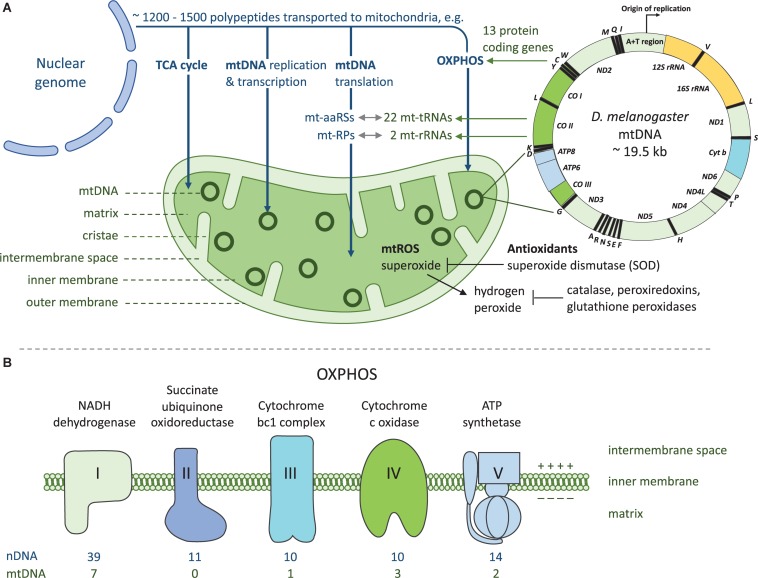
Mito-nuclear crosstalk in mitochondrial functions and energy production. **(A)** Mitochondria rely on coordinated functions of nuclear and mitochondrial genomes. Vast majority of the genes required for mitochondrial functions are encoded by the nuclear genome. These proteins are translated in the cytoplasmic compartment and transported into the mitochondrion post-translationally with a mitochondrial targeting signal. Products of both genomes are required for producing cellular energy in the form of ATP through OXPHOS. As a by-product of OXPHOS mitochondria produces reactive oxygen species (ROS) which are detoxified by antioxidants. mt-aaRSs, mitochondrial aminoacyl-tRNA synthetases; mt-RPs, mitochondrial ribosomal proteins. **(B)** OXPHOS takes place at the inner mitochondrial membrane and it comprises five enzyme complexes. Both nuclear and mitochondrial encoded proteins are required for OXPHOS complexes I and III-V, as complex II contains only nuclear encoded subunits. Complexes I-IV and two electron carriers form the respiratory chain which generates a proton gradient used by complex V to generate ATP.

Mito-nuclear crosstalk is required for mitochondrial functions. Mitochondria are cellular organelles of eukaryotic cells that are thought to have originated by endosymbiotic phagocytosis of an oxygen-converting α-proteobacterium by archaebacterium (19, 126). The primary function of mitochondria is to produce ATP through oxidative phosphorylation (OXPHOS) complexes I-V, and mitochondrial matrix is also the site of tricarboxylic acid cycle. Mitochondria contains multiple copies of a circular mtDNA (mtDNA copy number) distinct from that of the nuclear genome (Figure 1). Majority of the mtDNA genes required for aerobic energy production through OXPHOS have been shifted to the nuclear chromosomes and the remaining mitochondrial genome in most metazoans encodes for 37 genes, all crucial in OXPHOS. From these, 13 are polypeptide subunits of four of the five OXPHOS complexes, with the majority of the polypeptides encoded by nDNA (Figure 1B). Mitochondria contains its own translational system and the mtDNA encodes two rRNA and 22 tRNA genes as the mt-aaRS genes are encoded by the nuclear genome. Beside the 84 nDNA genes functioning in OXPHOS, around 1200-1500 nDNA encoded polypeptides are imported to and assembled within mitochondria, required for the various mitochondrial functions (Figure 1A). Nucleus and the mitochondria maintain a bidirectional regulation where the nuclear genome can signal to the mitochondria (anterograde signaling) for example to increase mitochondrial respiration. Mitochondria can signal (retrograde signaling) for example to induce cell death by releasing cytochrome c, or by controlling mitochondrial fusion and fission by AMP-activated protein kinase (57).

### Variation Arising From the Nuclear Genome

The vast majority of the proteins that are required for mitochondrial functions are encoded by the nuclear genome, translated in the cytosol and transported to mitochondria via mitochondrial targeting sequence which is removed upon entry into mitochondria. These proteins include the replication, transcription and translation machineries for mtDNA and the 84 polypeptide subunits needed for OXPHOS (summarized in Box 2). Mutations in nDNA directly affecting OXPHOS complex genes have been reviewed in (20). Maintenance genes of mitochondrial functions include regulatory genes of mitochondrial and cytosolic nucleotide pools to maintain balanced supply of mitochondrial dNTPs, involved with mtDNA nucleoid packaging, carrier proteins required for metabolite and cofactor transport across cellular and mitochondrial membranes, genes for mitochondrial lipid and membrane homeostasis, and mitochondrial fission/fusion and cristae organization [reviewed in (21)].

Mutations in mtDNA maintenance genes (replication and repair pathways and mtDNA nucleoid packaging) have been shown to cause mtDNA deletions, point mutations and even depletion (22). The most important mtDNA maintenance gene is the *DNA polymerase gamma* (*POLG*) which is responsible for the replication of the mtDNA. Almost 200 *POLG* mutations have been reported and these are the most common causes of mitochondrial disease. Mutations in *POLG* have been shown to cause large scale deletions and various other mutations to the mtDNA due to replication and/or repair machinery malfunctions and these have been connected to many mitochondrial diseases such as Alper’s syndrome, parkinsonism and multiple other neurodegenerative disorders (23). *POLG* was mutated in *Drosophila* to create a proofreading-deficient form resulting to drastically increased somatic mtDNA mutation frequency and mitochondrial dysfunction, which manifested as a shortened lifespan, a progressive locomotor deficit and a loss of dopaminergic neurons (24).

Regulation of OXPHOS gene transcription is tightly coordinated and must be able to establish efficient oxidative metabolism fulfilling the cell’s changing energy requirements. Components of the transcription machinery are encoded by the nuclear genome and have been reviewed in (25). Mossman et al., showed that in *D. melanogaster* cybrid lines the transcription of nuclear encoded mitochondrial genes were affected by mtDNA variation, indicating a retrograde signaling effect in transcription regulation (26). Hence, mutations in the transcription machinery can have a wide impact on the function of mitochondria, and variation in the mtDNA genes can affect the overall transcription efficiency of OXPHOS components, possibly also affecting their translation.

All proteins involved in mtDNA translation (27) are encoded by nuclear genes, involving ribosomal proteins, mt-aaRSs, tRNA modifying enzymes, and translation factors. Mutations in these genes have been shown to cause mitochondrial diseases due to dysfunction in the protein-synthesis machinery (27). mt-aaRSs are transported to mitochondria to catalyze an amino acid attachment to its complementary tRNA in aminoacylation reaction for translation of the thirteen mitochondrial proteins. All ribosomal RNAs and the transfer RNAs required for the translation of the mitochondrial proteins are encoded by the mtDNA (Figure 1). Hence, nuclear encoded components of the translation machinery need to be able to recognize mitochondrial counterparts for the production of mitochondrial proteins. mt-aaRS genes are central to cellular energy production and mutations in these can lead to variable disease phenotypes depending on the affected tissues and the energy demands of the cells in those tissue types (28). Mutations in both mt-tRNAs and mt-aaRSs can lead to disease and the clinical presentation has been shown to be highly specific to the affected mt-aaRS [reviewed in (29)]. However, diversity of pathologies is higher for mt-tRNA mutations than mt-aaRSs, possibly due to random distribution of heteroplasmic populations of mtDNA copies during mitotic segregation (28). In *Drosophila simulans* a variant of tyrosyl-tRNA synthetase interacts epistatically with a mitochondrially encoded *tRNA*^*Tyr*^ variant, leading to decrease in the activities of OXPHOS complexes I, III, and IV (30). At the organismal level this manifests as developmental delay, compromised bristle formation and decreased fecundity (30).

In *D. melanogaster*, a *tko* mutant (*tko^25*t*^*) carries a missense mutation in nuclear encoded mitoribosomal protein S12 causing a decrease in smaller ribosomal subunits. This causes decreased activity levels of all four OXPHOS complexes that contain mitochondrial encoded proteins (Figure 1), ultimately manifesting as developmental delay, temporary paralysis followed from vigorous shaking (bang sensitivity) and sensitivity to antibiotics and high sugar diet as well as impaired courtship behavior and hearing (31, 32). Similar defects have been found in fibroblasts of patients with antenatal encephalopathy caused by mutations in the *MRPS22* gene coding mitochondrial ribosomal protein, which result in a reduction of 12s rRNA (33).

### Variation Arising From the Mitochondrial Genome

The effect of the mitochondrial genome variation on innate immunity is intriguing as mtDNA does not follow the traditional Mendelian inheritance because it is inherited uniparentally through the maternal lineage. The mitochondrial genome contains 37 genes, from which 13 encode polypeptides, two rRNA genes and 22 tRNA genes (Box 2 and Figure 1A) and pathogenic mutations have been reported in all 37 mtDNA genes (34). mtDNA is subjected to much higher mutation rates than the nuclear genome and the mutation rate varies between species (35, 36). Besides mutations of the mtDNA replication and repair machinery, higher mutation rate of mtDNA is affected by the production of ROS within mitochondria as by-product of the OXPHOS. This can lead to a cycle in which ROS causes DNA damage, which in turn leads to dysregulation of respiration and accumulation of mutations.

Each cell contains numerous mitochondria and each mitochondrion may contain from a few to dozens of copies of mtDNA (the mtDNA copy number). Hence a given cell of a specific tissue type may contain thousands of copies of mtDNA and only one copy of nDNA. Unlike nDNA, mtDNA is not packed by histones but packed into protein-DNA complexes called nucleoids (37). *Drosophila* mtDNA contains one large A + T rich non-coding region (38), which in humans is called the D-loop. In both *Drosophila* and humans, the non-coding region is the starting point of mtDNA replication (39). Hence, variation in this region might cause changes in the mtDNA replication and have an effect on the mtDNA copy number content (40) and ultimately in energy production. mtDNA copy number is usually higher in tissues that have high energy demand such as brain, skeletal, and cardiac muscle tissues (41) and mtDNA mutations in these tissues can possibly lead to more pronounced phenotypes.

Besides heritable mtDNA mutations occurring in the germ line of the maternal lineage, the mtDNA of both females and males are subjected to spontaneous somatic mutations. mtDNA genetics is complicated due to its multi-copy nature. mtDNA mutations within a single cell (and even within a single mitochondria) can be either heteroplasmic due to a mix of mutated and wild type mtDNA, or homoplasmic where all mtDNAs contain either the mutated or the wild type form. In a heteroplasmic mutation the proportion of mutated mtDNA needs to exceed a certain threshold for the mutation to manifest. This can be due to the wild type mtDNA not being able to compensate the defect at that point. The threshold is also likely to be dependent on the mutation type and environmental effectors. Nuclear genotypes may also have variation in their ability to dampen or amplify the effects of specific deleterious mitochondrial mutations, which often demonstrate incomplete penetrance (42).

mtDNA mutations have been linked to various human diseases (43). In Leber hereditary optic neuropathy (LHON) the patient suffers from a loss of vision and the mtDNA mutations causing it are mostly considered homoplasmic (44). However, even though all the offspring of a homoplasmic mother inherits the LHON, only 50% of males and 10% of females develop the disease, showing that predicting the way the mtDNA mutation manifests, is difficult due to mito-nuclear crosstalk. Also, environmental factors can cause changes in the mtDNA mutation manifestation, as in the case of mtDNA encoded homoplasmic ribosomal RNA (RNR1) mutation that causes deafness early on in childhood. Specific antibiotics are associated with the manifestations of the clinical symptoms of the RNR1 mutation (45). Due to the complexity of the crosstalk of the two genomes and environmental factors, possible physiological compensations and the amplifying or dampening effects originating from nuclear genome variation and compatibility with the mtDNA, it is difficult to predict how the mtDNA mutations will eventually manifest themselves.

### Disruption of Mito-Nuclear Crosstalk

The optimal functioning of mitochondria relies on the correct transcription and translation of genes involved in respiration, and as mentioned above, these genes are found on both the nuclear and mitochondrial genomes. Several signaling pathways between the nucleus and mitochondria have been uncovered recently (46). Mutations in either nDNA or mtDNA have the potential to disrupt the crosstalk between mitochondrial and nuclear proteins, and can therefore disrupt efficient gene transcription and translation (30), with consequences for metabolic rates (47), aging (48) and sperm competitiveness (49), which ultimately have detrimental effects on organismal fitness (50). Evidence for these detrimental effects is especially clear in hybrids between closely related species or between divergent populations within species, where long-term coevolution between the nuclear and mitochondrial genomes has been broken up, resulting in novel combinations of nuclear and mitochondrial genomes (51, 52). For example, hybrids of the marine copepod *Tigriopus* show reduced activity of OXPHOS Complex IV (cytochrome c oxidase) because of the breakup of coevolved nuclear and mitochondrial-encoded subunits of the Complex IV (51). This mismatch between nuclear and mitochondrial genes is thought to be a strong selection pressure for the fixation of compensatory mutations in nuclear-encoded OXPHOS subunits (53).

Mito-nuclear interactions can also have strong effects on the outcome of infection. In *Drosophila*, a mito-nuclear incompatibility resulted in energetically compromised flies that were more susceptible to infection by a bacterial pathogen (54). Salminen et al., identified an OXPHOS Complex III mutation D_21_N in *D. melanogaster* mitochondrial *CYTB* gene, that was shown to cause larval stage melanotic nodules in a healthy nuclear background (6%) and a significant increase (56%) of melanotic nodules when the mitotype was introgressed into a *tko^25*t*^* nuclear background (55). Formation of melanotic nodules is considered a sign of activated cell mediated innate immunity, and it usually involves the proliferation and aggregation of hemocytes, *Drosophila* blood cells (56). Furthermore, *CYTB* mutation bearing mitotype in a *tko^25*t*^* nuclear background caused 100% pupal lethality, which is a first report of synthetic lethality between nuclear-mitochondrial interaction within a metazoan species (55).

## Mitochondrial Variation Can Affect Infection Outcome

The role of mitochondria in the response to infection is central, impacting multiple functions. First, intermediates of the mitochondrial tricarboxylic acid (TCA) cycle have a signaling function in innate immune responses. Second, mitochondria generate energy by producing ATP during OXPHOS (Box 2 and Figure 1B), and given the elevated energetic requirements of immunity, we may expect variation in mitochondrial functions to result in changes in ATP production, thereby generating heterogeneity in the response to infection. Third, mitochondrial metabolism may further promote protection against pathogens by producing ROS, with direct antimicrobial action. Finally, in mammalian models of immunity, damaged mtDNA has been shown to act as DAMP triggering inflammatory responses akin to those seen during infection. There is therefore increasing evidence that mitochondrial functions contribute to the host response to infection.

### Mitochondrial TCA Cycle Metabolites With Immune Signaling Functions

Mitochondrial tricarboxylic acid cycle (TCA, also called Krebs cycle and citric acid cycle) consists of a series of reactions where substrates originating from carbohydrates, fats and proteins have been fed into it and the metabolites from the cycle are transported into cytosol as building blocks for macromolecules or energy is released through the oxidation of acetyl-CoA. However, metabolites in the TCA cycle have also been shown to be involved in regulation of chromatin modifications, DNA methylation and post-translational modifications of proteins [reviewed in (57)]. Intermediates and derivatives of the TCA cycle have been shown to have non-metabolic signaling functions, in addition to their more conventional role as metabolites associated with bioenergetics (58). Non-metabolic functions of the TCA cycle intermediates succinate, itaconate, fumarate, 2-hydroxyglutarate and acetyl-CoA play a role in inflammation, and immune cell activation (58). For example, succinate is a pro-inflammatory metabolite as its production is enhanced during inflammation (59) and it acts as a signal from mitochondria to the cytosol to induce the expression of pro-inflammatory genes and increases the levels of antioxidant superoxide as a proinflammatory redox signal (60). Succinate has also been shown to accumulate in lipopolysaccharide treated macrophages (59). Itaconate on the other hand is endogenous protective and anti-inflammatory molecule that negatively regulates the inflammatory response and cytokine production (61–63) and also has direct antibacterial effects (64). TCA cycle intermediates have also been connected to epigenetic signaling (58). For example, fumarate has a role as an epigenetic inflammatory signal. Arts et al., showed that the accumulation of fumarate in immune activated monocytes was needed for trained immunity by enhancing cytokine production upon re-activation with lipopolysaccharide (65). Further, Acetyl-CoA has been shown to drive histone acetylation which can have profound impact on immune cell function (66). To summarize, mitochondrial variation may impact infection outcomes via their effect on TCA cycle products that have immune signaling functions.

### Changes in ATP Production

Mitochondria generate energy by producing ATP during OXPHOS (Box 2), and given the elevated energetic requirements of immunity, we may expect variation in mitochondrial function to result in changes in ATP production, thereby generating heterogeneity in the response to infection (54). Mutations in any of the nuclear or mitochondrial encoded OXPHOS complexes or in nuclear genes affecting replication, transcription or translation of mtDNA can affect the total electron transfer chain outcome, potentially causing a decrease in the total production of ATP. Severely decreased ATP synthesis is an obvious problem for cells with constant high energy demands such as cardiomyocytes and neurons (67) and decreased ATP synthesis can also increase the AMP/ATP ratio that can lead to activation of AMP-activated protein kinase and multiple signaling pathways (46). We might therefore expect mutations in nuclear or mitochondrial encoded components that cause a reduction in ATP to result in a decrease of immune cell function.

### Role of ROS in Immune Responses

Reactive oxygen species are a group of reactive molecules and free radicals derived from molecular oxygen which are now known to have a role in cellular homeostasis (68). Elevated levels of ROS can cause oxidative stress, cellular-, and DNA damage in eukaryotic cells. Mutations in nuclear or mitochondrial genes encoding the protein subunits of OXPHOS complexes I and III can cause a decrease or an increase in ROS production, depending on the mutation. One of the most evident roles of mitochondrial functions in innate immunity is the production of ROS by leakage from mitochondrial ETC. The majority of ROS are produced during mitochondrial ETC (mtROS), and some by oxidoreductase enzymes such as NADPH oxidase, a multicomponent membrane bound enzyme complex. Common ROS include superoxide (O^–^_2_), hydrogen peroxide (H_2_0_2_), hydroxyl radical (OH), hydroxide ion (OH^–^) and nitric oxide (NO). Prolonged oxidative stress is harmful and so detoxification of ROS via scavenging enzymes and antioxidants is vital. Therefore, mutations in the nuclear encoded antioxidants that are targeted to detoxify ROS, can also have an impact on immune response. Antioxidant superoxide dismutase (SOD) is transported to mitochondria where it converts superoxide to hydrogen peroxide. Hydrogen peroxide outside mitochondria is converted to water and oxygen with the help of catalase, peroxiredoxins and glutathione peroxidases (Figure 1).

mtROS is produced in all cell types that contain mitochondria and it has been connected to regulation of signaling pathways (69), apoptosis (70), inflammation (71), cellular adaptation to hypoxia (72), cellular differentiation (73), and autophagy (74). In addition to the regulative role of mtROS, a growing body of evidence has highlighted the role of ROS as a target of regulation of immune signaling pathways (14, 75). mtROS serves several roles within both the humoral and cell-mediated arms of innate immunity, including direct elimination of pathogens through its microbicidal effects. However, it is still unclear what is the exact mechanism of the bactericidal effect of mtROS upon bacterial infection as the effect seem to be the type of ROS and pathogen specific [reviewed in (68)] and aligns with the increased heterogeneity of the innate immune response.

Alongside its role in promoting bacterial clearance, mtROS also functions in signaling for hemocyte proliferation and differentiation in *Drosophila* and has been identified as an essential signaling molecule in *Drosophila’s* cellular immune response to parasitoid infection (76). ROS plays a likely role as a key signaling molecule within the *Drosophila* lymph gland as it has been suggested to prime the quiescent hemocyte progenitors within the lymph gland for differentiation (77). It has been shown that reduction of ROS significantly retards progenitor differentiation whereas upregulation of ROS via OXPHOS Complex I disruption produces a phenotype with a significantly higher hemocyte population (77).

Transmitochondrial cell lines can be created by combining enucleated cells that contain the mtDNA of interest with cells that lack their mtDNA (78). Data obtained from transmitochondrial cell lines suggest that mtDNA variants on a controlled nuclear background can alter ROS levels of cells (79). Organismal cybrids have also been shown to differ in OXPHOS parameters (34) and effect of mtDNA variation on altered ROS production has been studied in *Drosophila in vivo*, showing that specific mtDNA variants can elevate ROS production (80).

### Cytosolic mtDNA as a Danger Signal

During infection, elevated levels of ROS can cause mitochondrial and cellular damage resulting mtDNA leakage. Due to the evolutionary origin of mitochondria it harbors resemblance to bacterial DNA making it appear non-self. mtDNA is surrounded by a double-membrane structure and membrane damage could lead to leakage of mtDNA outside mitochondria and elicit self-derived immune activation. When mtDNA is located outside mitochondria in the cytoplasm of the cell or in extracellular space, it can trigger immune responses by directly engaging the host’s innate immune pattern-recognition receptors (PRRs) [reviewed in (81)]. PRRs are conserved receptors that recognize viral, bacterial and fungal particles as well as molecules released from injured cells. The release of mtDNA outside of mitochondria can occur from dying cells, during injury, cellular stress or infection, and mtDNA outside of mitochondria engages PRRs and functions as damage-associated molecular pattern (DAMP) leading to enhancement of pro-inflammatory responses (71). In mammalian models of immunity, inflammasomes are innate immune related signaling complexes that monitor the cytosolic compartment of the cell and are involved in the secretion of cytokines upon infection and recognition of DAMPs (81). Altered mitochondrial dynamics, production of mROS and release of mtDNA outside mitochondria have all been linked to inflammasome activation (82). In mammalian system extracellular circulatory mtDNA has been shown to act as an endogenous Toll-like receptor TLR9 agonist and been connected to many TLR9- dependent inflammatory diseases (81). Cytosolic mtDNA (83) as well as cytosolic double-stranded RNA created during bidirectional transcription of mtDNA (84) has been shown to trigger antiviral responses in human.

## Using *Drosophila* to Study Mitochondrial Variation and Mito-Nuclear Interactions in Immunity

Mitochondrial function and the content of the mitochondrial genome are highly conserved among metazoans e.g., between humans and the fruit fly *D. melanogaster* and the latter has been widely used to model human mitochondrial diseases [reviewed in (85)]. Besides studying the naturally occurring variation of nuclear genes affecting mitochondrial function, i.e., mtDNA replication, transcription and translation as well as polypeptides needed for TCA and OXPHOS, it is possible to exploit the highly sophisticated genetic toolbox that exists for *D. melanogaster.* With the binary gene expression systems, such as GAL4-UAS, one can modify the expression of a desired gene within a specific tissue within a specific time, providing a route to investigate the effect of specific genes in chosen tissues on a given phenotype (86). For example, overexpression and gene knock-down methods could allow the modification of the gene expression of nuclear encoded genes that are transported to mitochondria, hence altering the mitochondrial function, assuming that the mitochondrial import stage does not dampen the effect of genetic modification.

**FIGURE 2:**
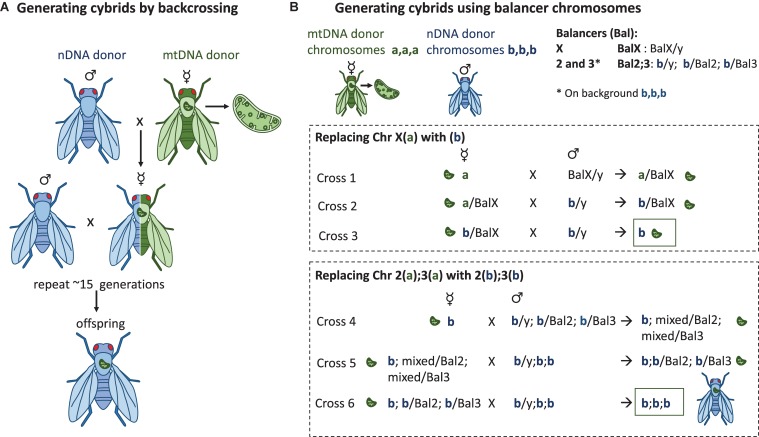
Methods to generate *Drosophila in vivo* cybrid lines. **(A)** Repeated backcrossing. The chosen mtDNA variant is added during the first cross where the virgin females from the mtDNA donor strain are crossed with the males of the nuclear donor strain. Virgin females of the following progeny are crossed again with the males of the nuclear donor strain. This will be repeated for >15 generations, after which the cybrid progeny contain the wanted mtDNA variant on a nuclear background that in theory is the same as the one in the nuclear donor strain. This method also has the potential to reveal possible mito-nuclear epistasis during the course of the backcrossing. **(B)** Balancer chromosome method. *Drosophila* males do not go through recombination, whereas in females the recombination can be controlled with the balancer chromosomes which do not recombine with the normal chromosome homologs during meiotic prophase. The presence of balancer chromosomes in the progeny can be recognized by the dominant marker mutations that the balancers carry, e.g., mutations affecting eye shape, body color, or wing morphology. If the progeny do not have the balancer, it has the normal homolog of the wanted chromosome. Three (labeled here a,a,a or b,b,b) of the four *D. melanogaster* chromosomes can be replaced to contain the genetic content of the wanted nuclear donor strain by using the balancer chromosome method. The mtDNA from the maternal donor strain is introgressed to the strain during the first cross and the chromosome content is replaced chromosome at a time by using the correct progeny of the previous cross. For clarity, chromosomes 2 and 3 have not been marked to the cross where the X(a) is replaced with the X(b).

Another approach to study mitochondrial variation is to focus on naturally occurring variation in mtDNA. Investigating the effect of mtDNA mutations is complicated by cross-talk between the mitochondria and the nucleus of a cell, with nuclear genes generally responsible for controlling mitochondrial activity. With the cytoplasmic hybrid, aka. cybrid model, specific mtDNAs can be introgressed onto controlled nuclear backgrounds, making it possible to focus on the effects arising from the mitochondrial genome (Figure 2). It is presently not possible to genetically target and modify the gene expression of specific mtDNA genes, and so the cybrid model relies on using natural mtDNA variants found through different genetic screens. However, there are methods for creating random mutations to mtDNA genome such as using *POLG* mutants (87, 88) or to more specific regions with targeted restriction enzymes (89).

By generating transmitochondrial cybrid cells, it is possible to study the effects of mtDNA variation at the cellular level (78). This *in vitro* approach has been used previously to investigate the cellular effects of mtDNA polymorphism associated with an aggressive form of breast cancer (90). Similarly, Bellizzi et al., hypothesized that the transcription of stress-responder nuclear genes can be modulated according to the mtDNA variability. They showed that osteosarcoma cells depleted of their own mitochondria and repopulated with different ones, modulated the expression of cytokines and cytokine receptors due to the variability of the mtDNA (91).

The effects of mtDNA variation at the organismal level may vary significantly from what can be inferred from cellular level. The transmitochondrial *in vitro* method is limited in investigating the impact of mtDNA variation affecting an entire organism, for example upon environmental stress, limited diet or when fighting infection. However, *D. melanogaster* offers a feasible way of creating and utilizing cybrid strains for *in vivo* experiments of the effects of mtDNA variation at the organismal level (26, 30, 50), as well as quantifying the effects of mtDNA, nDNA and their interaction. Given the ease with which flies can be sampled from natural populations, options of screening the mtDNA variation by sequencing and crossing and breeding the lines in constant laboratory conditions, *Drosophila* cybrid lines offer a unique opportunity to disentangle variation in immunity arising from mtDNA polymorphism from the more commonly investigated variation in nuclear-encoded genes associated with canonical immune pathways.

There are three ways of creating cybrids and two of them, repeated backcrossing and using balancer chromosomes, are common methods in *Drosophila*, explained in detail below and in Figure 2. In the third method, Mitochondrial Replacement Therapy (MRT), mtDNA is introduced directly to a novel nuclear environment. In MRT the nucleus of a mtDNA mutation bearing female is transferred to an enucleated egg of a mitochondrially healthy donor (92). To date, MRT has been performed in two other mammalian species beside humans, in macaques (93) and mice (94, 95).

### Generating *Drosophila* Cybrids by Repeated Backcrossing

Because mtDNA is inherited uniparentally through the mother, the chosen donor mtDNA can be introgressed onto wanted nuclear background by multiple generations of backcrossing (Figure 2A). In the first cross the virgin females containing the donor mtDNA are crossed with males containing the wanted nuclear background, resulting in progeny with 100% maternal mtDNA, 50% maternal nDNA and 50% paternal nDNA. This is followed by a series of crosses where the virgin females of each generation are crossed with males of the nuclear donor strain. With each cross, the proportion of paternal nDNA increases by 50% from the previous generation, meaning that in theory, after 10 generations of backcrossing, less than 0.1% of the maternal nDNA should be present. An important caveat to introgression by repeated backcrossing is that although in theory only 10 generations should be required to obtain a line over 99.9% of the paternal nuclear genome, in practice this value will be lower due to strong linkage disequilibrium between loci that have a close genetic distance (called linkage drag) (96). From a mapping perspective this linkage can be advantageous, but the backcrossing approach has the potential to select for compatible nuclear partners, masking true incompatibilities (also lethal combinations). The number of generations of backcrossing required to break linkage between two loci will therefore depend on the genetic distance such that the number of backcrosses *N* = *Log*(*1-r*)(*1-P*) where *r* is the recombination frequency, and *P* is the probability of separation (97). For example, for two loci separated by a 20 cM interval, at least 17 generations of backcrossing are required to have a 95% certainty of a crossover event (97). However, even after multiple generations of backcrossing, some loci are likely to never recombine if they result in strongly deleterious or even lethal phenotypes, and such loci will remain a source of residual heterozygosity regardless of the number of generations of backcrossing.

This method has been used in *D. melanogaster* experiments to study the effect of mito-nuclear interactions on mtDNA copy number, respiration, development time and weight (40) as well as in experiments where the effect of mtDNA variants on mitochondrial diseases has been assayed in combination with the balancer chromosome method (55, 98). The method has been also employed in other insect species where the use of balancer chromosomes is not possible, where the effect of mito-nuclear crosstalk has been studied on traits such as metabolism and aging in *D. simulans* (99), metabolic rate in *Drosophila subobscura* (100), and personality (101), bioenergetics, aging, life history traits (102), and male mating costs (47) in seed beetles.

### Utilizing *Drosophila* Balancer Chromosomes

*Drosophila* has four chromosomes that include the X/Y sex chromosome pair and autosomal chromosomes 2, 3, and 4. The fourth chromosome is very small, contains only around 80 genes (103) and does not recombine. The genetic tool box developed for *D. melanogaster* allows the replacement of the entire chromosomes with the wanted content of specific strains or mutation. This is done with the help of balancer chromosomes. Balancer chromosomes (104) are multiply inverted and scrambled chromosomes that are not able to undergo crossover with their normal chromosome homologs. They also contain genetic markers that enable the recognition of their segregation. As the small chromosome *4* does not go through crossing over there are no balancers designed for this chromosome. Nuclear genomes can be constructed by replacing the chromosomes in multiple crosses in a properly planned crossing scheme, and eventually controlling the nuclear genome (Figure 2B). For creating *Drosophila* lines that contain the wanted mtDNA variant on a controlled nuclear background, the first cross includes the mtDNA donor females after which the wild-type chromosomes will be replaced one by one with the wanted isogenic chromosomes [crossing scheme explained e.g., in (30, 105, 106)]. This method has been utilized to study the effect of mtDNA variation and mito-nuclear interaction on aging (105), sex differences in aging, respiration and fertility (107, 108), starvation resistance, lipid proportion and physical activity (106) as well as ROS production and mtDNA copy number (109).

## Conclusion and Future Perspectives

*Drosophila* has contributed significantly for our understanding of mitochondrial variation in both mitochondrial diseases and in immunity. Here we have highlighted strengths of this experimental powerhouse and described approaches that will link these two important fields to address the question how mitochondrial variation and specifically mtDNA variation affect innate immune functions. This is significant because a number of mitochondrial mutations have been associated with increased susceptibility to infection in humans (110, 111) and recent genetic screens have revealed vast variation in *Drosophila* mtDNA (112). The *Drosophila* model will also be relevant in the context of mitochondrial replacement therapy as a tool to test for potential incompatibilities that may result from specific mito-nuclear combinations (113). Given the homology between vertebrate and invertebrate innate immunity (114), the *Drosophila* model has the translational potential to generate novel candidate genes originating from mitochondrial sources of disease susceptibility and resistance, and for development of new therapeutic targets.

## Author Contributions

TS and PV wrote the manuscript.

## Funding

TS was supported by an Academy of Finland Fellowship (grants 322732 and 32879). PV was supported by a Leverhulme Trust Research Project Grant RPG-2018-369, a Branco Weiss Fellowship (https://brancoweissfellowship.org/), and a Chancellor’s Fellowship (School of Biological Sciences, University of Edinburgh).

## Conflict of Interest

The authors declare that the research was conducted in the absence of any commercial or financial relationships that could be construed as a potential conflict of interest.

## Abbreviations

AMP: antimicrobial peptide
ATP: adenosine triphosphate
DAMP: danger-associated molecular pattern
ETC: electron transport chain
IMD: immune deficiency
mt-aaRS: mitochondrial aminoacyl-tRNA synthetase
mtDNA: mitochondrial DNA
mtROS: mitochondrial reactive oxygen species
nDNA: nuclear DNA
NF- κ B: nuclear factor kappa-light-chain-enhancer of activated B cells
OXPHOS: oxidative phosphorylation
ROS: reactive oxygen species.
